# Molecular Diagnosis of *Pseudoterranova decipiens* Sensu Stricto Infections, South Korea, 2002‒2020

**DOI:** 10.3201/eid2806.212483

**Published:** 2022-06

**Authors:** Hyemi Song, Seungwan Ryoo, Bong-Kwang Jung, Jaeeun Cho, Taehee Chang, Sooji Hong, Hyejoo Shin, Woon-Mok Sohn, Jong-Yil Chai

**Affiliations:** Korea Association of Health Promotion, Seoul, South Korea (H. Song, S. Ryoo, B.-K. Jung, J. Cho, T. Chang, S. Hong, H. Shin);; Gyeongsang National University College of Medicine, Jinju, South Korea (W.-M. Sohn);; Seoul National University College of Medicine, Seoul (J.-Y. Chai)

**Keywords:** Pseudoterranova, molecular diagnosis, Pseudoterranova decipiens sensu stricto, parasites, nematodes, infections, anisakiasis, foodborne infections, food safety, zoonoses, South Korea

## Abstract

Human *Pseudoterranova decipiens* larval infections were diagnosed by molecular analysis of mitochondrial *cox*1 and *nd*1 genes in 12 health check-up patients in South Korea during 2002–2020. Based on high genetic identity (99.3%–100% for *cox*1 and 96.7%–98.0% for *nd*1), we identified all 12 larvae as *P. decipiens* sensu stricto.

Human anisakiasis, which is caused by infection with larvae of the family Anisakidae after consuming infested marine fish or squids, is one of the most serious foodborne zoonotic diseases (*1*). Several species of *Anisakis* (*A. simplex* sensu stricto, *A. physeteris*, and *A. pegreffii*) (*1**–**3*), *Pseudoterranova* (*P. decipiens* sensu stricto, *P. azarasi*, and *P. cattani*) (*4**–**6*), and *Contracecum* (*C. osculatum*) (*7*) nematodes have been reported to cause human infections.

Human anisakiasis was reported in the Netherlands during 1960 and has been found to occur in various parts of the world, including Japan and South Korea (*1*). Most human case-patients were infected with larvae of *A. simplex* s.s. (*1*). However, after 1999, a considerable number of cases infected with *A. pegreffii* nematodes (a sibling species of *A. simplex* s.s.) were diagnosed in Italy, Japan, and South Korea on the basis of molecular analysis of the larvae (*2**,**3*). Compared with *Anisakis* spp. nematodes, human infections with *Pseudoterranova* spp. nematodes have been relatively rare in Asia (*1**,**4**–**6*). In South Korea, among 645 anisakidosis cases recorded after 1971 until 2015, only ≈11.8% were infected with *Pseudoterranova* larvae (*8*). However, all of these *Pseudoterranova* infections were diagnosed on the basis of only the morphology of the larvae (*8*).

Within the genus *Pseudoterranova*, 8 species have been validated on the basis of molecular and morphologic/biologic characteristics: *P. decipiens* s.s., *P. kogiae*, *P. ceticola*, *P. azarasi*, *P. krabbei*, *P. bulbosa*, *P. decipiens* E, and *P. cattani* (*9*). Among those, 6 species (*P. decipiens* s.s., *P. krabbei*, *P. bulbosa*, *P. azarasi*, *P. decipiens* E, and *P. cattani*) are morphologically and biologically related to each another and designated as the *P. decipiens* species complex or *P. decipiens* sensu lato (*9*). These species can be discriminated by allozyme or molecular genetic analyses (*10*).

In our study, 12 human pseudoterranoviasis cases were found among patients who visited health check-up centers or hospitals in South Korea during 2002–2020 because of vague abdominal discomfort. Larvae were extracted by using gastrointestinal endoscopy (11 case-patients) or colonoscopy (1 case-patient). The larvae were confirmed to be *P. decipiens* s.s. by sequence analysis of the mitochondrial cytochrome *c* oxidase 1 (*cox*1) and NADH dehydrogenase subunit 1 (*nd*1) genes.

The patients consisted of 5 men (41–55 years of age) and 7 women (29–59 years of age). A total of 12 larvae (1 larva from each patient) were collected from the stomach (11 patients) or cecum (1 patient) (Appendix Table 1) and were processed for sequencing of 2 mitochondrial genes (Appendix).

Sequences of the *cox*1 (141 bp) (samples nos. OK539788–OK539799) and *nd*1 (153 bp) genes (OK539800–OK539807) showed high homologies with the sequences of *P. decipiens* s.s. (GenBank accession no. NC_031645 for *cox*1 and *nd*1). The homology between samples from this study and *P. decipiens* s.s. was 99.3%–100% for *cox*1 and 97.4%–98.0% for *nd*1 (Appendix Tables 1–3).

The phylogenetic tree for *cox*1 showed that the 12 study samples were tightly clustered with *P. decipiens* s.s. reported from Germany but separate from *P. bulbosa* from Canada, *P. cattani* from Chile, *P. krabbei* from Norway, and *P. azarasi* from Japan (Figure). The phylogenetic tree for *nd*1 showed that 8 study samples were closely aligned with *P. decipiens* s.s. reported from Germany but clearly separated from *P. cattani* from Chile, *P. bulbosa* from Canada, *P. krabbei* from Norway, and *P. azarasi* from Japan (Figure). We also determined genetic distances between the study specimens and *P. decipiens*, *P. azarasi*, *P. bulbosa*, *P. cattani*, and *P. krabbei* for *cox1* (Appendix Table 2) and *nd*1 (Appendix Table 3).

**Figure:**
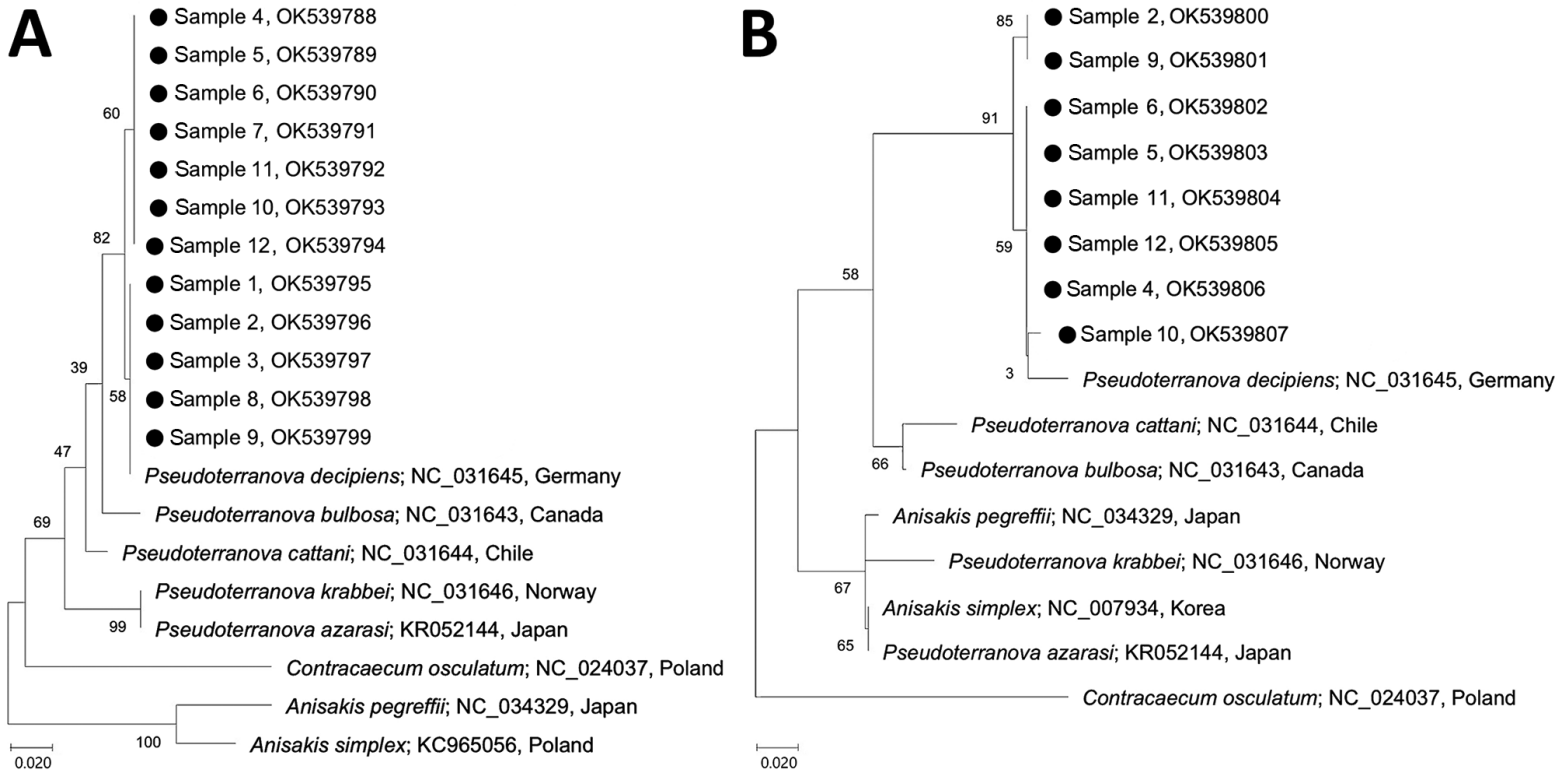
Phylogenetic analyses of *Pseudoterranova* nematode larvae extracted from 12 health check-up patients in South Korea, 2002–2020 (black dots), in comparison with other anisakid species. A) mitochondrial cytochrome oxidase c gene sequences; B) mitochondrial NADH dehydrogenase gene sequences. Trees were constructed by using the neighbor-joining method based on the Kimura 2-parameter model of nucleotide substitution with 1,000 bootstrap replications and viewed by using MEGA-X (https://www.megasoftware.net). GenBank accession numbers and country of origin are provided for reference sequences. Details of patient information for the 12 samples from this study are provided in Appendix Table 1). Numbers along branches are bootstrap values. Scale bars indicate nucleotide substitutions/site.

For the specific diagnosis of anisakid larvae, analysis of the larval morphology is highly useful. However, extracting a fully intact larva from human patients for high-quality morphologic analysis is usually difficult. In such instances, molecular analysis of the larvae is helpful and essential for obtaining a specific diagnosis. Analyses of the internal transcribed spacer region and partial 28S rDNA could discriminate *P. decipiens* s.s. from *P. bulbosa*, *P. krabbei*, *P. cattani*, and possibly *P. decipiens* E (*10*). However, great sequence similarity was observed between *P. decipiens* s.s. and *P. azarasi*. Thus, it was difficult to distinguish them by using nuclear genes (*10*). Some investigators used mitochondrial genes, including *cox*1, *cox*2, and *nd*1, to distinguish them (*4**,**5*).

In our study, we used 2 mitochondrial genes, *cox*1 and *nd*1, to distinguish the species of *Pseudoterranova*. Our results showed that the nematode specimens from these patients nested within *P. decipiens* s.s. but were clearly separated from *P. azarasi*, *P. bulbosa*, *P. cattani*, and *P. krabbei* samples available in GenBank. Molecular analysis of larvae will be useful for obtaining specific diagnoses of infection.

Appendix Additional information on molecular diagnosis of *Pseudoterranova decipiens* sensu stricto infections, South Korea, 2002‒2020.
