# Supplementary material for: Molecular Diagnosis of Pseudoterranova decipiens Sensu Stricto Infections, South Korea, 2002‒2020
Source: Emerg Infect Dis. 2022 Jun;28(6):1283–5. doi: 10.3201/eid2806.212483 (PMC9155879; doi:10.3201/eid2806.212483)
Supplement: Appendix — Additional information on molecular diagnosis of Pseudoterranova decipiens sensu stricto infections, South Korea, 2002‒2020. [file 21-2483-Techapp-s1.pdf]

Article DOI: https://doi.org/10.3201/eid2806.212483

Molecular Diagnosis of *Pseudoterranova decipiens* Sensu Stricto Infections, South Korea, 2002–2020

Appendix

Methods

Larvae preserved in 70% ethanol (3 cases), 10% formalin (1 case), or mounted in glycerin jelly after clearing with glycerin–alcohol (8 cases) were processed for molecular analysis of the mitochondrial genes, in particular, cytochrome *c* oxidase 1 (*cox*1) and NADH dehydrogenase subunit 1 (*nd*1). Genomic DNA was isolated by using the DNeasy Blood and Tissue Kit (QIAGEN, https://www.qiagen.com) according to the manufacturer's instructions.

Based on the complete mitochondrial genome of *Pseudoterranova decipiens* (GenBank accession no. NC_031645), specific pairs of primers were designed by using the tool of the National Library of Medicine (https://www.nlm.nih.gov). The *cox*1 primers used were Pse_*cox*1_F (5′-TGCTGGTTTACACGGTTTTCC-3′) and Pse_*cox*1_R (5′-CGATGA CCCACAAAAGACTCC-3′). The *nd*1 primers were Pse_*nd*1_F (5′-TATTAGGTGGCA GTCAGCAGC-3′) and Pse_*nd*1_R (5′-AAAAGACCCCCGGAACCAAAA-3′). The thermal cycling profile was denaturation at 95°C for 5 min, 35 cycles of denaturation at 95°C for 20 s, annealing at 61°C (*cox*1) or 62°C (*nd*1) for 20 s, and extension at 72°C for 1 min with a final extension at 72°C for 5 min.

DNA sequencing was performed at Macrogen Co. Ltd. (https://www.macrogen.com). Phylogenetic trees were constructed by using the neighbor-joining method and viewed by using the MEGA-X program (https://www.megasoftware.net).

Appendix Table 1. Characteristics of 12 case-patients who had *Pseudoterranova decipiens* larvae infection diagnosed*

Case-patient	Year detected	Age, y/sex	Infection site	Sample storage	Morphologic features of larva, IC/BT/M	% Identity for *cox*1/*nd*1†
1	2002	45/F	Stomach	GJ mount	+/NO/+	100/–
2	2002	45/F	Stomach	GJ mount	+/+/+	100/96.7
3	2003	55/M	Stomach	GJ mount	NO/NO/NO	100/–
4	2004	54/F	Stomach	GJ mount	+/+/+	99.3/98.0
5	2005	39/F	Stomach	GJ mount	+/+/+	99.3/98.0
6	2005	59/F	Stomach	GJ mount	+/+/+	99.3/98.0
7	2008	46/M	Stomach	GJ mount	+/+/NO	99.3/–
8	2010	48/F	Cecum	GJ mount	NO/NO/NO	100/–
9	2015	29/F	Stomach	70% ethanol	NO/+/+	100/96.7
10	2016	48/M	Stomach	10% formalin	+/+/+	99.3/97.4
11	2018	54/M	Stomach	70% ethanol	NO/NO/+	99.3/98.0
12	2020	41/M	Stomach	70% ethanol	+/+/+	99.3/98.0

*BT, boring tooth; GJ, glycerin jelly; IC, intestinal cecum; M, mucron; NO, Not observable because of partial destruction of larva. –, negative; +, positive.
†For *cox1*, GenBank accession no. is NC_031645; for *nd1*, GenBank accession no. is NC_031645.

Appendix Table 2. Genetic distances shown as % differences of *cox*1 sequences (141 bp) among specimens of *Pseudoterranova* larvae

Sample no. and name	1	2	3	4	5	6	7
1 OK539788~OK539794 (this study)							
2 OK539795~OK539799 (this study)	0.7						
3 *P. decipiens* s.s. NC_031645 (Germany)	0.7	0.0					
4 *P. azarasi* KR052144 (Japan)	6.3	7.0	7.0				
5 *P. bulbosa* NC_031643 (Canada)	3.5	2.8	2.8	6.3			
6 *P. cattani* NC_031644 (Chile)	2.8	3.5	3.5	4.9	4.9		
7 *P. krabbei* NC_031646 (Norway)	6.3	7.0	7.0	0.0	6.3	4.9	

Appendix Table 3. Genetic distances shown as % differences of *nd1* sequences (153 bp) among specimens of *Pseudoterranova* larvae

Sample no. and name	1	2	3	4	5	6	7	8
1 OK539800~OK539801 (this study)								
2 OK539802~OK539806 (this study)	1.3							
3 OK539807 (this study)	2.0	0.7						
4 *P. decipiens* s.s. NC_031645 (Germany)	3.3	2.0	2.6					
5 *P. azarasi* KR052144 (Japan)	13.7	13.7	14.4	15.7				
6 *P. bulbosa* NC_031643 (Canada)	8.5	8.5	9.2	9.2	7.8			
7 *P. cattani* NC_031644 (Chile)	10.5	10.5	11.1	11.1	10.5	2.6		
8 *P. krabbei* NC_031646 (Norway)	16.3	16.3	17.0	18.3	3.3	9.8	7.2	
